# A Comparison of Normalization Techniques for Individual Baseline-Free Estimation of Absolute Hypovolemic Status Using a Porcine Model

**DOI:** 10.3390/bios14020061

**Published:** 2024-01-23

**Authors:** Tamara P. Lambert, Michael Chan, Jesus Antonio Sanchez-Perez, Mohammad Nikbakht, David J. Lin, Afra Nawar, Syed Khairul Bashar, Jacob P. Kimball, Jonathan S. Zia, Asim H. Gazi, Gabriela I. Cestero, Daniella Corporan, Muralidhar Padala, Jin-Oh Hahn, Omer T. Inan

**Affiliations:** 1 The Wallace H. Coulter Department of Biomedical Engineering, Georgia Institute of Technology, Atlanta, GA 30332, USA; mchan81@gatech.edu (M.C.); omer.inan@ece.gatech.edu (O.T.I.); 2 School of Electrical and Computer Engineering, Georgia Institute of Technology, Atlanta, GA 30332, USA; jasp@gatech.edu (J.A.S.-P.); mohnikbakht@gatech.edu (M.N.); dlin91@gatech.edu (D.J.L.); anawar3@gatech.edu (A.N.); sbashar7@gatech.edu (S.K.B.); gcestero3@gatech.edu (G.I.C.); 3 The Donald P. Shiley School of Engineering, University of Portland, Portland, OR 97203, USA; kimballj@up.edu; 4 Division of Neurology & Neurological Sciences, Stanford School of Medicine, Palo Alto, CA 94304, USA; jzia@stanford.edu; 5 John A. Paulson School of Engineering and Applied Sciences, Harvard University, Allston, MA 02134, USA; agazi@schmidtsciencefellows.org; 6 Structural Heart Research and Innovation Laboratory, Carlyle Fraser Heart Center, Emory University Hospital Midtown, Atlanta, GA 30308, USA; daniella.corporan@gmail.com (D.C.); psmuralidhar@gmail.com (M.P.); 7 Division of Cardiothoracic Surgery, Emory University School of Medicine, Atlanta, GA 30322, USA; 8 Department of Mechanical Engineering, University of Maryland, College Park, MD 20742, USA; jhahn12@umd.edu

**Keywords:** machine learning, wearable biosensors, noninvasive biosensors, continuous monitoring, hypovolemia, digital biomarkers, decompensation

## Abstract

Hypovolemic shock is one of the leading causes of death in the military. The current methods of assessing hypovolemia in field settings rely on a clinician assessment of vital signs, which is an unreliable assessment of hypovolemia severity. These methods often detect hypovolemia when interventional methods are ineffective. Therefore, there is a need to develop real-time sensing methods for the early detection of hypovolemia. Previously, our group developed a random-forest model that successfully estimated absolute blood-volume status (ABVS) from noninvasive wearable sensor data for a porcine model (*n* = 6). However, this model required normalizing ABVS data using individual baseline data, which may not be present in crisis situations where a wearable sensor might be placed on a patient by the attending clinician. We address this barrier by examining seven individual baseline-free normalization techniques. Using a feature-specific global mean from the ABVS and an external dataset for normalization demonstrated similar performance metrics compared to no normalization (normalization: R^2^ = 0.82 ± 0.025|0.80 ± 0.032, AUC = 0.86 ± 5.5 × 10^−3^|0.86 ± 0.013, RMSE = 28.30 ± 0.63%|27.68 ± 0.80%; no normalization: R^2^ = 0.81 ± 0.045, AUC = 0.86 ± 8.9 × 10^−3^, RMSE = 28.89 ± 0.84%). This demonstrates that normalization may not be required and develops a foundation for individual baseline-free ABVS prediction.

## 1. Introduction

Hypovolemia is a physiological condition referring to the depletion of extracellular fluid volume in the body [1]. This condition can be categorized as absolute hypovolemia, where a reduction occurs in the total intravascular blood volume due to conditions such as hemorrhage [2,3], or relative hypovolemia, where an increase in vascular capacitance leads to a reduction in vascular preload and limited blood circulation in the extremities due to conditions such as sepsis [2,4]. If left unchecked, hypovolemia can lead to hypovolemic shock, a life-threatening condition that leads to organ injury and multiorgan failure [5]. In addition to health ailments, hypovolemia is of great concern for the military, professional athletes, firefighters, and other professions that require significant physical exertion or are at increased risk for injury resulting in fluid loss [6,7,8].

The body can lose up to 10% of the total blood volume with little change to cardiac output and/or arterial blood pressure [5,9]. Once the body achieves 10% blood loss or higher, the body’s vascular physiology begins to change, leading to changes in the vital signs. For example, diastolic blood pressure increases, resulting in a narrowed pulse pressure and an elevation in mean arterial blood pressure (MAP) [5]. A loss of blood greater than 10% also leads to a reduction in cardiac preload, reducing cardiac output which subsequently reduces tissue perfusion and oxygen availability [9]. Between 15 and 30% loss of the total blood volume leads to more dangerous consequences, and if blood loss persists, leads to hypovolemic shock. Systolic blood pressure begins to decline, along with an increase in heart rate, or tachycardia [9,10]. At this point, the oxygen supplies received by the vital organs are no longer sufficient for the body, inducing anaerobic metabolism. This creates acidic conditions in the body due to lactic acidosis [11], causing the body to prioritize blood flow to vital organs. Without timely intervention and treatment, conditions such as lactic acidosis can be further exacerbated, resulting in multiorgan failure and death [5].

Despite the well-documented systemic compensation for hypovolemia, detecting it in real time remains a challenge. This is due to differences in individual tolerance levels of hypovolemia among people prior to cardiovascular collapse, and because the changes in vital signs are typically very late in the process [8]. Thus, it is difficult to find early signs of worsening hypovolemic conditions. Currently, there is no gold standard for detecting hypovolemia [12,13]. Obtaining an accurate estimate of blood volume is critical to providing lifesaving medical treatment when treating acute medical conditions, such as hemorrhage due to physical trauma or blood-volume loss due to severe dehydration [4,9,13,14]. This estimate is essential for treating hypovolemia, as providing too much fluid resuscitation can lead to edema and impair oxygen diffusion while providing too little fluid resuscitation can lead to the progression of hypoxia and organ damage [15,16]. Hypovolemia is currently diagnosed by clinical examination, including measuring a myriad of nonspecific physiological markers such as elevated heart rate, hypotension, and dry mucous membranes, in addition to biomarkers such as sodium content in urine and blood gas measurements. However, this method can be inaccurate in the absence of relevant clinical context, yielding low sensitivity and specificity. Vital signs in particular can be highly dependent on factors such as age and pre-existing conditions [12]. Vital signs also demonstrate unreliability due to the body’s compensatory mechanisms. They often do not demonstrate the true extent of the patient’s condition until the patient has an advanced decompensation status such as hypovolemic shock [5,10,17], greatly diminishing the effect of lifesaving treatment [18].

Given the current limitations in estimating ABVS, wearable technology demonstrates the potential to provide clinicians with a noninvasive, readily available, sensitive, and specific way to assess ABVS in real time using physiological waveforms. The compensatory reserve measure (CRM), a method that assesses changes in arterial waveform morphology, demonstrates promise as a method to accurately predict ABVS in real time in combination with wearables [19,20]. This method has been validated previously for predicting hypovolemia in human subjects [20,21,22] but still presents challenges among certain populations such as the military and those with health conditions that damage the peripheral vasculature. The CRM method relies on volume-clamping-based finger-cuff blood pressure and transmissive photoplethysmogram (TPPG), which are not suitable for those who have sustained catastrophic injuries to their fingers, those in a cold environment that restricts blood flow to the fingers, or those with damaged peripheral vasculature which render the signals unusable due to the presence of artifacts [23,24,25,26].

**Figure 1:**
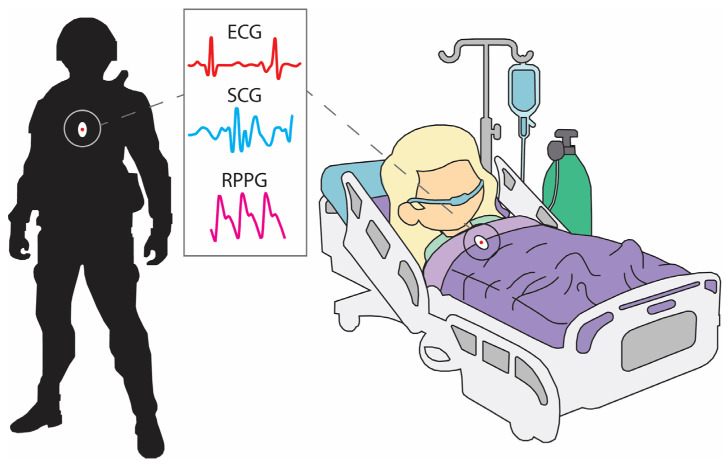
Accurately estimating blood-volume status is critical for soldiers in direct combat or patients at high risk of decompensating due to hemorrhage. Blood-volume status estimation is not only relevant for determining the fluid loss but also for determining how much fluid resuscitation the patient requires. A wearable device fixed to the chest (circled in the figure above) may be able to measure cardiac signals (electrocardiogram (ECG), seismocardiogram (SCG), and reflective photoplethysmogram (RPPG)) indicative of blood-volume status in real time.

Based on these limitations, dependence on one sensor or signal may render the operator unable to assess ABVS and provide an incomplete picture of the person’s health status. Therefore, there is much utility in using multimodal sensing to estimate ABVS [27]. In the previously published literature, electrocardiogram (ECG), seismocardiogram (SCG), and reflective photoplethysmogram (RPPG) sensors have not only demonstrated the ability to be mounted onto a chest-worn patch [28] but demonstrate the ability to provide a reliable estimate for ABVS [17,29] and to classify hypovolemia into absolute vs. relative hypovolemia [17]. Our work builds on top of previously published work that uses features extracted from ECG, SCG, and RPPG (Figure 1) to develop a random-forest model able to predict the blood-volume status during relative hypovolemia, absolute hypovolemia, and resuscitation [17,29]. Although these models demonstrated measurable success in their ability to accurately predict ABVS and decompensation status, these models require access to the patient’s baseline ABVS data, which may not be available in emergency situations. Our work proposes several methods of normalization to estimate ABVS without individual baseline ABVS measurements using absolute ABVS data and multimodal sensing dependent on cardiac performance. We hypothesized that only the inclusion of the features most relevant to ABVS, and the inclusion of the demographic parameter weight during normalization, would lead to improved ABVS estimates compared to no normalization, thereby eliminating the need for the individual baseline to compute ABVS.

We analyzed seven different normalization techniques that can be leveraged for the real-time estimation of ABVS status in hypovolemia. Our work has several innovative aspects, including (1) a comparison of seven individual baseline-free normalization techniques to scale ABVS data, (2) a reduced feature model to improve ABVS estimation accuracy, (3) the use of demographic data to normalize ABVS data, and (4) the use of a myocardial infarction dataset to provide an accurate individual baseline-free normalization of the ABVS data. These contributions will help to inform the development of wearable technology that can track these conditions in real time, allowing clinicians to administer life-saving treatments in a timely manner. Our work demonstrates, for the first time to our knowledge, the ability to estimate ABVS using features extracted from ECG and SCG waveforms without the presence of a patient’s baseline measurements. This work demonstrates that accurate ABVS estimation can occur in the absence of individual baseline normalization, indicating that individual baseline normalization may not be required. This work also demonstrates the ability to provide reliable estimates of ABVS using a reduced feature model (4-feature model compared to the original 12-feature model).

## 2. Materials and Methods

### 2.1. Data Collection and Feature Extraction

#### 2.1.1. ABVS Dataset

**Figure 2:**
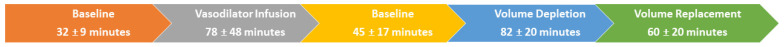
Timeline of the experimental protocol for the induction of relative and absolute hypovolemia. The timeline includes the standard deviation of the time that it took to conduct each section of the experiment. This study was conducted by the Georgia Institute of Technology.

ECG, SCG, and RPPG data were collected from six Yorkshire swine, all of which underwent a protocol approved by the Institutional Animal Care and Use Committees of the Georgia Institute of Technology (protocol A100276 approved 04/10/18), Translational Testing and Training Labs Inc. (T3 Labs; protocol GT48P approved 04/23/18) and the Department of the Navy Bureau of Medicine and Surgery (BUMED). The baseline characteristics for each pig are included below in Table 1:

The protocol proceeded as follows in Figure 2; relative hypovolemia was induced by the administration of nitroglycerine starting at 10–20 mcg/min. Doses were increased by 5–10 mcg/min until the pig reached cardiovascular collapse (a 20% decrease in MAP) or the pig received a dose of 500 mcg/min. Absolute hypovolemia was induced by hemorrhaging the pigs at 7% increments of the total blood volume determined using the Evan’s Blue dye protocol and allowing the cardiovascular system to stabilize for 5–10 min after hemorrhage. This procedure ceased at cardiovascular collapse, and the animals were resuscitated at 7% increments, pausing for 10–15 min between increments to allow for cardiovascular stabilization. The pigs were anesthetized using Xylazine and Telazol. This state was maintained using inhaled isofluorane during mechanical ventilation. All pigs were terminated at the end of the experiment by intravenous injection of potassium chloride or by exsanguination.

Each animal was monitored invasively and noninvasively. Invasive monitoring occurred via the catheterization of the carotid artery, capturing aortic pressure (AoP), femoral artery pressure (FAP), right atrial pressure (RAP), and pulmonary capillary wedge pressure (PCWP) using vascular introducers. Cardiac output and PWCP were measured by the Edwards 131F7 Swan Ganz catheter (Edwards Lifesciences Corp., Irvine, CA, USA) enabled by the embedded thermistor. ADInstrumentsMLT0670 pressure transducers (ADInstruments Inc., Colorado Springs, CO, USA) were connected to the vascular introducers via pressure monitoring lines. Catheter data were continuously acquired using an ADInstruments Powerlab 8/35 acquisition system with a sampling frequency of 2 kHz.

Noninvasive monitoring occurred via ECG, SCG, and RPPG wearable systems. ECG data were collected using a three-lead configuration and a BIOPAC ECG100C amplifier (BIOPAC Systems Inc., Goleta, CA, USA). SCG data were collected using an ADXL354 accelerometer placed midsternum (Analog Devices Inc., Norwood, MA, USA) and a BIOPAC HLT100c transducer interface module. RPPG data were gathered using a transreflectance transducer (BIOPAC TSD270) positioned over the femoral artery and a BIOPAC OXY200 veterinary pulse oximeter. All signals were filtered using finite impulse-response zero-phase digital band pass filters with a Kaiser window. The cutoff frequencies were as follows: 0.5 to 40 Hz for the ECG, 1 to 40 Hz for the SCG, and 0.5 to 10 Hz for the RPPG [17]. All signals were captured at a sampling frequency of 2 kHz and were connected to the BIOPAC MP160. The following twelve features were extracted from the previous study.

(1) *ECG features:* heart rate (HR) and three measures of HR variability (HRV; the time-domain heart-rate variability (SDRR), HRV using the Poincaré method (SD1/SD2), and the frequency-domain HRV (LF/HF)) [17]. Rationale: indicates cardiovascular stress [17];
(2) *SCG features:* left ventricular ejection time (LVET). Rationale: exhibits strong correlations with measures of ventricular function. Additional information is included in [17];
(3) *RPPG features:* RPPG amplitude (PPGamp) and pleth variability index (PVI). Rationale: Typically estimates blood pressure, fluid responsiveness, and vascular tone [17];
(4) *Cross-domain features:* the following features required more than one signal to compute: pre-ejection period (PEP), PEP/LVET, pulse arrival time (PAT), HR-normalized PAT (nPAT), and pulse transit time (PTT). Rationale: (1) exhibits strong correlations with measures of ventricular function (PEP and PEP/LVET) and (2) estimate blood pressure, fluid responsiveness, and vascular tone (PAT, nPAT, and PTT) [17].

#### 2.1.2. Myocardial Infarction Dataset

From a separate study, ECG, SCG, and RPPG data were collected from 18 Yorkshire swine, all of which underwent a protocol to induce myocardial infarction approved by the T3 Labs Institutional Animal Care and Use Committee (IACUC) and by a Designated Member Review in accordance with federal regulations. Data were collected by a chest-mounted wearable patch called the Seismopatch at a frequency of 1 kHz. The data was collected at baseline prior to the induction of the myocardial infarction. The Seismopatch, fabricated at Northwestern University (Evanston, IL, USA), contained a combination of ECG, SCG, and RPPG sensors, and was validated in prior work [28]. The following features were extracted to normalize the corresponding features from the ABVS Dataset: HR, PEP, LVET, and PEP/LVET. Features from the RPPG signal were not extracted due to signal corruption, and, therefore, were excluded from the study. For this reason, the analyses involving the hypovolemia and myocardial infarction dataset only included the ECG and SCG features.

### 2.2. Normalization Methods

Data normalization is a critical pre-processing step to achieving accurate results when evaluating machine-learning models. Normalization involves scaling or transforming the data into a specific range, thus improving model performance when performing a machine-learning or classification problem [30,31]. The three most-common normalization methods are z-score, min–max, and decimal-scaling normalization [32,33]. Z-score normalization involves normalizing the data by subtracting the mean and dividing it by the standard deviation. Min–max normalization scales all datapoints between 0 and 1 using the minimum and maximum feature values. Decimal-scaling normalization normalizes the data by using a power of 10 to scale the features. Several normalization methods were evaluated to determine the feasibility of accurate ABVS estimation without normalizing it to individual baseline measurements.

Our methods utilize more traditional normalization methods [31] in combination with some modifications to normalize the pig data. For our work, we used (1) z-score normalization and (2) subtracting and dividing by the mean as the foundation for our seven normalization methods. For our initial preliminary analysis, we performed a Spearman correlation analysis between the data normalized using the original baseline normalization method and the common normalization methods. We evaluated correlations using the Spearman correlation method as our data satisfied several assumptions, including being on an interval scale and having pairwise observations and a monotonic relationship. Spearman’s correlations were computed rather than Pearson’s correlations because linear regressions produced residuals that did not satisfy the homoscedasticity assumption of Pearson’s correlations. We performed the normalization feature by feature and correlated the feature data from the original baseline normalization technique with the data normalized using the common normalization methods. We hypothesized that the method exhibiting the strongest correlation to the original baseline normalized data would perform the best for our model. Although the differences in performance between the three most-common normalization methods in our preliminary analysis were negligible, Z-score normalization was chosen as a method because it scales the features to comparable distributions. A summary of the results is in Table 2.

**Figure 3:**
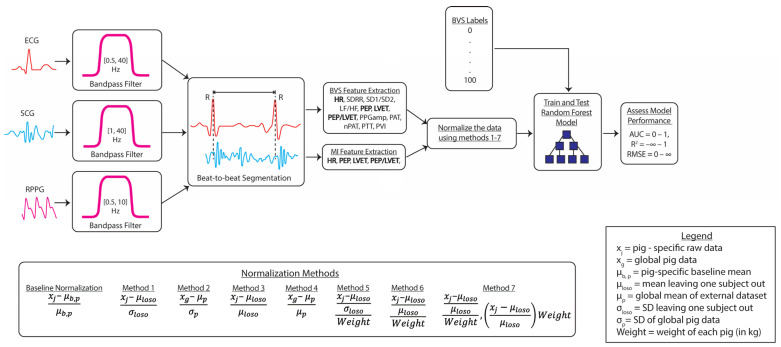
A block diagram representation of the signal-processing methods used to extract signal features and normalize feature data to train and test the random-forest model. Model performance was assessed using the area under the curve (AUC), the coefficient of determination (R^2^), and the root mean square error (RMSE). The external dataset refers to the myocardial infarction dataset.

Subtracting and dividing by the mean to obtain the percentage change of the ABVS values relative to the baseline were included as another method, given its successful deployment in the prior literature for normalizing ABVS features [17,29]. Using these two methods, we developed three additional normalization methods, including the demographic feature and the weight of the pig for comparison. Additionally, two of the normalization methods implement parameters extracted from an external dataset that did not undergo blood-volume loss to normalize the ABVS data. This was done to determine the feasibility of achieving accurate ABVS estimation using non-ABVS data. Data during the 7% incremental blood draws was not included in the analysis. Only the data during the 5–10 min cardiovascular stabilization period were used for normalization [17]. We normalize the feature data after feature extraction, which is presented in Figure 3. Our normalization methods test (1) the ability of the data to be normalized without using individual baseline data (methods 1–7), (2) the ability to normalize the data using a global mean and standard deviation from an external dataset (methods 2 and 4), (3) using weight as a parameter for normalization (methods 5–7), and (4) using mixed methods for normalization (method 7). Below, each normalization method is explained in detail:

Method 1: Z-score normalization using the leave-one-subject-out (LOSO) method. The feature data are normalized by subtracting the mean and dividing it by the standard deviation of each feature from the ABVS dataset. To ensure that the data are not biased, we normalize each pig’s feature data (held-out data) with the mean and standard deviation of the other pigs (held-in data). We repeat this process with each subsequent pig until the feature data of all pigs are normalized.

Method 2: using Z-score normalization, we normalize the features from the ABVS dataset by subtracting the global mean of each feature from the myocardial infarction dataset and dividing it by the standard deviation of the myocardial infarction dataset.

Method 3: using the normalization method reported in [17], we normalize the features from the absolute ABVS dataset using the LOSO method, as reported in Method 1. We normalize the features by subtracting the mean and dividing by the mean of each feature from the ABVS dataset. To ensure that the data are not biased, we normalize each pig’s feature data (held-out data) with the mean of the other pigs (held-in data). We repeat this process with each subsequent pig until the feature data of all pigs are normalized.

Method 4: using the normalization method reported in [17], we normalize the features from the ABVS dataset by subtracting the global mean of each feature from the myocardial infarction dataset and dividing it by the mean of the myocardial infarction dataset.

Method 5: the same as Method 1, except for Method 1′s normalization being divided by the weight of the pig. Weight was included as a variable due to its relationship with blood volume. Blood volume generally increases in direct proportion with an increase in weight. However, the type of tissue that the weight gain is attributable to influences the strength of this relationship. This is because adipose tissue is not as vascularized and, therefore, not as readily perfused by blood as muscle tissue, which impacts blood volume and may ultimately impact ABVS [34]. Due to this reason, we hypothesized that weight would be an influential factor in normalizing the ABVS data and included it as a parameter.

Method 6: the same as Method 3, except for Method 3′s normalization being divided by the weight of the pig.

Method 7: feature-specific normalization—the literature suggests that it may be more advantageous to use different normalization methods to normalize different features rather than one normalization method for all features [31]. Therefore, we applied different normalization methods to different features to determine if our ABVS estimate improves. This method is a combination method of Method 3 being multiplied by the pig’s weight, and Method 6. The features that were normalized using Method 3 multiplied by the pig’s weight were HR and LVET, and the features that were normalized using Method 6 were PEP and PEP/LVET. This method was determined by examining multiple combinations of the methods above to determine which combination of methods yielded data with the highest correlation to the respective feature data from the original individual baseline normalization method. After testing multiple options, the normalization combination with the highest correlation was Method 3 and Method 6 as described above.

### 2.3. Training and Testing the Model

#### 2.3.1. Model Label Assignment

Labels for each pig were created depending on how much blood loss they were able to tolerate before decompensation. Recalling the methods in the Section 2.1.1, increments of 7% of blood were withdrawn from the pig until the pig reached cardiovascular collapse (a 20% decrease in mean arterial pressure (MAP)). Therefore, one pig had 2 blood loss steps, creating discrete labels of 0%, 50%, and 100%, three pigs had 3 blood loss steps, creating discrete labels of 0%, 33%, 67%, and 100%, and two pigs had 4 blood loss steps, creating discrete labels of 0%, 25%, 50%, 75%, and 100%. These labels, along with the absolute ABVS data, were used to train and test the random-forest model developed in our previous study to predict ABVS levels using the features previously discussed in the Section 2.1.1. The receiver operating characteristic (ROC) curve was determined by labeling 100% ABVS as fully decompensated, or 1, and labeling all other ABVS percentages as 0, indicating that the pigs were not fully decompensated [17].

#### 2.3.2. Model Evaluation and Function

Our work used the random-forest model as outlined in [17]. The models contained an ensemble of 200 decision trees trained with bagging and a depth of 10 consecutive splits in accordance with prior work [17]. Class weights were included during training for the purpose of normalizing the number of heartbeats between the pigs and at each ABVS level. LOSO-CV was used to evaluate the model that had the greatest generalizability to subjects not seen in the training set. The model for absolute hypovolemia was first assessed with 12 features (HR, SDRR, SD1/SD2, LF/HF, PEP, LVET, PEP/LVET, PPGamp, PAT, PTT, nPAT, and PVI) with and without the individual baseline normalization method reported in [17] to establish the baseline model performance metrics (R^2^, AUC, and RMSE). Due to RPPG signal corruption in the myocardial infarction dataset, we were unable to extract RPPG features from this dataset. Given that one of our goals was to normalize features from the ABVS dataset using the myocardial infarction dataset, we decided to remove RPPG signals from both datasets and run our models using only the ECG and SCG features (HR, SDRR, SD1/SD2, LF/HF, PEP, LVET, PEP/LVET). We examined our models using a combination of the ECG and SCG features outlined in Table 3. Prior work revealed that the ECG and SCG-related features demonstrated the most importance when estimating ABVS [17,27]. Our goal was to test the model using the ECG and SCG-related features present in the ABVS and myocardial infarction datasets to determine if the model would yield similar or superior results to the 12-feature model. We first assessed multiple models with ECG and SCG features, with and without the normalization method reported in [17] to establish a baseline performance for the models. Each model was evaluated 10 times to ensure repeatability of the results, and the average of R^2^, AUC, and RMSE for each trial is reported in Table 3. Our initial assessment revealed that a four-feature model containing HR, PEP, LVET, and PEP/LVET, normalized using the individual baseline, yielded superior results compared to all other tested models (Table 3 and Table S1). Due to these results, we proceeded to analyze our results only using the four-feature model without measures of heart-rate variability.

## 3. Results

**Figure 4:**
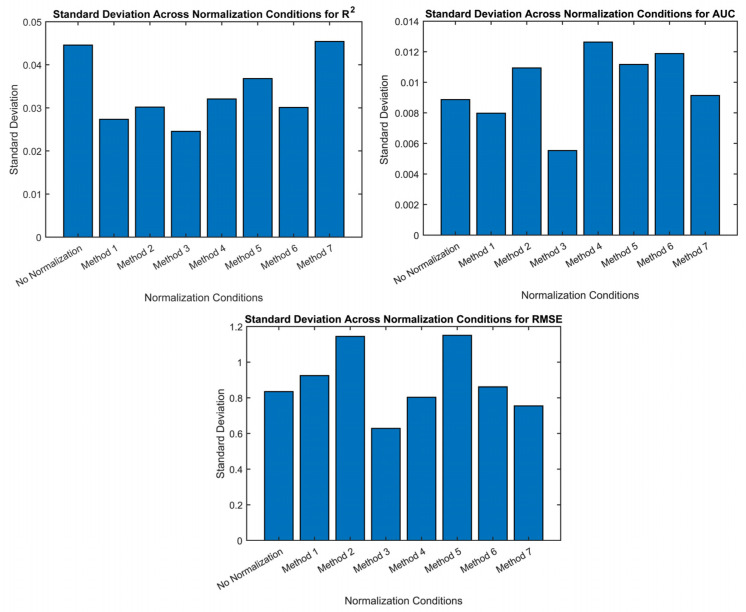
Standard deviations across all metrics (R^2^, RMSE, and AUC). Method 3 demonstrates the lowest standard deviation across all metrics, indicating the most consistent performance among all seven methods for 10 trials.

We implemented normalization Methods 1–7 on the ABVS dataset. Our results are presented below in Table 4:

The results demonstrate that the normalization methods do not significantly improve the results compared to having no normalization method present and, therefore, indicate that normalization may not be required to obtain accurate ABVS estimation. Looking across all metrics, the normalization methods achieved similar performance to having no normalization present. There were marginal improvements in the RMSE for the normalization methods compared to having no normalization method present. Method 3 demonstrated the potential for a slight reduction in variability given its consistently lower standard deviation across all metrics, as demonstrated in Table 4 and Figure 4, indicating consistent results across multiple trials. Methods 2 and 4 reveal that using a feature-specific mean and standard deviation from an external dataset for normalization has a minimal impact on the results. The model that yielded the best overall performance was the four-feature model with individual baseline normalization, even when compared to the original individual baseline model, indicating that a leaner model containing the features most important to ABVS estimation may yield more accurate results [17,29].

## 4. Discussion

This work evaluated several individual baseline-free estimation techniques to determine the feasibility of accurate ABVS estimation in the absence of the individual pig’s baseline blood-volume status. Our results suggest that normalizing to other participants’ data does not notably improve ABVS estimation compared to no normalization, although all methods, including having no normalization present, achieved accurate ABVS estimation. All methods displayed similar performance metrics and a slight reduction in measurement variability, indicating that normalization of the data may not be required at all. Although these methods do not outperform the original 12-feature model or the 4-feature model with individual baseline normalization, these models serve as a starting point to indicate the possibility of achieving accurate ABVS estimation in the absence of the individual’s baseline data and normalization. The results also suggest that, if a practitioner wanted to optimize their model for a reduction in variability of their R^2^, AUC, and RMSE estimates, they may want to consider normalizing their data using only the global mean of other subjects with the same disease pathology, as demonstrated in Method 3.

Our analysis demonstrated that a 4-feature model outperformed the original 12-feature model. Our analysis also reveals that one-, two-, and three-feature models with baseline normalization and PEP/LVET, HR, and LVET as features were still able to provide an accurate estimate of ABVS. These observations may be due in part to the four features (HR, PEP, LVET, and PEP/LVET) being among the most important features to estimate BVS according to the prior literature [17,29]. This may also be due to the four features being measures of central vascular function compared to peripheral vascular function. During events that compromise blood flow such as exposure to cold temperatures or blood loss, the body prioritizes blood flow to the vital organs and restricts blood flow to the extremities [10,35,36]. Since the pigs were undergoing blood loss in our protocol, this may have some impact on the predictive capabilities of features that measure peripheral vascular function. Blood flow may be restricted to the central vasculature in cases of severely compromised peripheral vascular function, which may demonstrate the benefit of only using the ECG and SCG features. Given that we were able to achieve improved results using only the ECG and SCG-based features, these results imply that if a PPG sensor were to malfunction or if the patient was experiencing poor peripheral vascular function, an accurate estimate of ABVS can still be made using ECG- and SCG-related features. These results also indicate that we may also be able to reduce the number of sensors and features used and still obtain an accurate ABVS estimation, potentially leading to a leaner model requiring less computation time, similar to the model found in [37]. This implies the ability of our model to make accurate ABVS estimates if a PPG sensor is unavailable or if the patient has peripheral circulatory pathologies, moving our model one step closer to a real-time estimation of ABVS.

Our analysis had several limitations. There was a small sample size of pigs who underwent the ABVS protocol (*n* = 6), so to determine the translatability of the results, we will have to test our model on a larger sample size. Another limitation is that the proposed normalization methods are not time-series normalization methods and, therefore, may not account for the changes that are happening within the various ABVS levels. Therefore, we can potentially consider time-series normalization methods such as adaptive normalization, which was used to normalize wind-speed data as a function of time for future work [38]. Our model relied on features that were among the most important in the prior work, which may have introduced bias. Future work should either use a different machine-learning model or an absolute blood-volume loss dataset to verify results. When including weight as a parameter in our model, we did not determine if the weight of the pig was primarily influenced by muscle tissue or adipose tissue due to data on the height of the pigs being unavailable. Given that these tissue types impact blood flow differently, future work should include measures that assess body-fat content, such as body mass index, and determine its influence on blood loss. An additional limitation is that, although the external dataset includes data from pigs who were undergoing surgery to induce myocardial infarction, the data were taken at baseline before any procedure commenced and, therefore, are representative of healthy individuals. Given that those experiencing ABVS may have a variety of pre-existing health conditions, future work should include feature data from pigs with different pathological conditions to determine if a global mean and standard deviation from these datasets are effective parameters for individual baseline-free normalization using a random-forest model.

## Figures and Tables

**Table 1:** Summary baseline characteristics for each pig.

Subject	1	2	3	4	5	6
Sex	CM	CM	CM	F	F	F
Age (days)	150	131	118	118	133	114
Weight (kg)	71.4	65.4	54.5	60	66.5	60.5
HR (bpm)	84	59	70	67	79	93
CO (L/min)	4.5	4.2	2.9	4.3	4.9	5.8
Est. Blood Volume (L)	4.6	4.3	3.5	4.3	3.3	3.3
AoP S/D (mmHg)	90/45	93/52	104/56	72/32	83/34	88/49

HR, heart rate; CO, cardiac output; AoP, aortic arch pressure; S/D, systolic and diastolic; CM, castrated male; F, female.

**Table 2:** Correlations for each feature between the original baseline normalization method and common normalization method data. *p*-values were determined at the 95% confidence level.

Normalization Methods	Features							
	HR		PEP		LVET		PEP/LVET	
	ρ	*p*	ρ	*p*	ρ	*p*	ρ	*p*
**Z-Score**	0.90	0	0.57	0	0.94	0	0.57	0
**Min–Max**	0.90	0	0.57	0	0.94	0	0.57	0
**Decimal Scaling**	0.90	0	0.57	0	0.94	0	0.57	0

**Table 3:** Preliminary ABVS model results assess the initial 12-feature model versus models using only ECG and SCG features. The metrics below (R^2^, AUC, and RMSE) represent the mean across 10 trials for each normalization method. *** Indicates the model with the best performance. IBN = Individual baseline normalization.

Method Name	Features	Method Equation	R^2^	AUC	RMSE (%)
12-feature model w/IBN	HR, SDRR, SD1/SD2, LF/HF, PEP, LVET, PEP/LVET, PPGamp, PAT, PTT, nPAT, and PVI	$\frac{x_{j}-\mu_{b,p}}{\mu_{b,p}}$	0.86	0.89	21.20
12-feature model w/o IBN	HR, SDRR, SD1/SD2, LF/HF, PEP, LVET, PEP/LVET, PPGamp, PAT, PTT, nPAT, and PVI		0.91	0.89	29.02
1-feature model w/IBN	PEP/LVET	$\frac{x_{j}-\mu_{b,p}}{\mu_{b,p}}$	0.82	0.81	22.31
1-feature model w/o IBN	PEP/LVET		0.25	0.58	40.39
2-feature model w/IBN	PEP/LVET, HR	$\frac{x_{j}-\mu_{b,p}}{\mu_{b,p}}$	0.84	0.88	22.10
2-feature model w/o IBN	PEP/LVET, HR		0.78	0.83	34.32
2-feature model w/IBN	PEP/LVET, SDRR	$\frac{x_{j}-\mu_{b,p}}{\mu_{b,p}}$	0.78	0.87	23.92
2-feature model w/o IBN	PEP/LVET, SDRR		0.83	0.82	34.53
3-feature model w/IBN	PEP/LVET, HR, and LVET	$\frac{x_{j}-\mu_{b,p}}{\mu_{b,p}}$	0.86	0.90	21.25
3-feature model w/o IBN	PEP/LVET, HR, and LVET		0.92	0.84	29.39
3-feature model w/IBN	PEP/LVET, SDRR, and LVET	$\frac{x_{j}-\mu_{b,p}}{\mu_{b,p}}$	0.78	0.87	23.82
3-feature model w/o IBN	PEP/LVET, SDRR, and LVET		0.78	0.85	35.39
4-feature model w/IBN ***	HR, PEP, LVET, and PEP/LVET	$\frac{x_{j}-\mu_{b,p}}{\mu_{b,p}}$	0.90	0.92	19.47
4-feature model w/o IBN	HR, PEP, LVET, and PEP/LVET		0.81	0.85	28.96
4-feature model w/IBN	HR, SDRR, LVET, and PEP/LVET	$\frac{x_{j}-\mu_{b,p}}{\mu_{b,p}}$	0.80	0.89	23.07
4-feature model w/o IBN	HR, SDRR, LVET, and PEP/LVET		0.92	0.87	33.36
5-feature model w/IBN	HR, SDRR, PEP, LVET, and PEP/LVET	$\frac{x_{j}-\mu_{b,p}}{\mu_{b,p}}$	0.86	0.91	21.24
5-feature model w/o IBN	HR, SDRR, PEP, LVET, and PEP/LVET		0.90	0.88	30.09
6-feature model w/IBN	HR, SDRR, SD1/SD2, PEP, LVET, and PEP/LVET	$\frac{x_{j}-\mu_{b,p}}{\mu_{b,p}}$	0.84	0.89	22.32
6-feature model w/o IBN	HR, SDRR, SD1/SD2, PEP, LVET, and PEP/LVET		0.94	0.89	28.77
7-feature model w/IBN	HR, SDRR, SD1/SD2, LF/HF, PEP, LVET, and PEP/LVET	$\frac{x_{j}-\mu_{b,p}}{\mu_{b,p}}$	0.83	0.89	22.73
7-feature model w/o IBN	HR, SDRR, SD1/SD2, LF/HF, PEP, LVET, and PEP/LVET		0.91	0.89	30.22

*x_j_*, pig-specific raw data; *µ_b,p_*, pig-specific individual baseline mean.

**Table 4:** ABVS model results for normalization Methods 1–7. All models contained only 4 features (HR, PEP, LVET, and PEP/LVET). The mean (M) and standard deviation (SD) were calculated for 10 trials for each normalization method.

Method Name	Method Equation	R^2^		AUC		RMSE (%)	
		M	SD	M	SD	M	SD
No normalization		0.81	0.045	0.86	8.9 × 10^−3^	28.89	0.84
Method 1	$\frac{x_{j}-\mu_{loso}}{\sigma_{loso}}$	0.82	0.027	0.86	8.0 × 10^−3^	27.88	0.92
Method 2	$\frac{x_{g}-\mu_{p}}{\sigma_{p}}$	0.80	0.030	0.86	0.011	28.41	1.14
Method 3	$\frac{x_{j}-\mu_{loso}}{\mu_{loso}}$	0.82	0.025	0.86	5.5 × 10^−3^	28.30	0.63
Method 4	$\frac{x_{g}-\mu_{p}}{\mu_{p}}$	0.80	0.032	0.86	0.013	27.68	0.80
Method 5	$\frac{\frac{x_{j}-\mu_{loso}}{\sigma_{loso}}}{Weight}$	0.82	0.037	0.86	0.011	27.54	1.15
Method 6	$\frac{\frac{x_{j}-\mu_{loso}}{\mu_{loso}}}{Weight}$	0.81	0.030	0.86	0.012	27.66	0.86
Method 7	$\frac{\frac{x_{j}-\mu_{loso}}{\mu_{loso}}}{Weight}$ $\left(\frac{x_{j}-\mu_{loso}}{\mu_{loso}}\right)Weight$	0.80	0.045	0.86	9.1 × 10^−3^	27.90	0.75

*x_j_*, pig-specific raw data; *x_g_*, global pig data; *µ_loso_*, mean leaving one subject out; *µ_p_*, global mean of the external dataset; *σ_loso_*, standard deviation (SD) leaving one subject out; *σ_p_*, global SD of the external dataset; Weight, weight of each pig (in kg).

## Data Availability

All data needed to evaluate the conclusions in the paper are present in the paper and/or the Supplementary Materials. Additional data related to this paper may be requested from the authors. The data collected and analyzed in this study for the Hypovolemia Dataset are publicly available on IEEE DataPort at https://ieee-dataport.org/open-access/wearable-and-catheter-based-cardiovascular-signals-during-progressive-exsanguination (accessed on 15 January 2024). The data collected and analyzed in this study for the Myocardial Infarction Dataset can be made available upon request.

## Supplementary Materials

The following are available online at https://www.mdpi.com/article/10.3390/bios14020061/s1: Figure S1: Preliminary performance of ABVS models, Table S1: Graphical illustration of preliminary performance of ABVS models.
