# Supplementary material for: A Comparison of Normalization Techniques for Individual Baseline-Free Estimation of Absolute Hypovolemic Status Using a Porcine Model
Source: Biosensors (Basel). 2024 Jan 23;14(2):61. doi: 10.3390/bios14020061 (PMC10886994; doi:10.3390/bios14020061)
Supplement: Supplementary file 1 [file biosensors-14-00061-s001.zip › biosensors-2759088-supplementary.pdf]

A Comparison of Normalization Techniques for Individual Baseline-Free Estimation of Absolute Hypovolemic Status using a Porcine Model
Supplementary Material

Table S1. Preliminary performance of ABVS models, including mean (M) and standard deviation (SD). **Indicates the model with the best performance. IBN = Individual baseline normalization.

**Method Name**	**Features**	**Method Equation**	**R^2**		**AUC**		**RMSE** (%)	
			**M**	**SD**	**M**	**SD**	**M**	**SD**
12-feature model w/ IBN	HR, SDRR, SD1/SD2, LF/HF, PEP, LVET, PEP/LVET, PPGamp, PAT, PTT, nPAT, and PVI	$\frac{x_j - \mu_{b,p}}{\mu_{b,p}}$	0.86	0.012	0.89	$5.5*10^{-3}$	21.20	0.81
12- feature model w/o IBN	HR, SDRR, SD1/SD2, LF/HF, PEP, LVET, PEP/LVET, PPGamp, PAT, PTT, nPAT, and PVI		0.91	0.017	0.89	0.012	29.02	1.05
1-feature model w/ IBN	PEP/LVET	$\frac{x_j - \mu_{b,p}}{\mu_{b,p}}$	0.82	$3.8*10^{-4}$	0.81	$2.3*10^{-4}$	22.31	$9.9*10^{-4}$
1-feature model w/o IBN	PEP/LVET		0.25	$1.7*10^{-3}$	0.58	$6.5*10^{-4}$	40.39	0.027
2-feature model w/ IBN	PEP/LVET, HR	$\frac{x_j - \mu_{b,p}}{\mu_{b,p}}$	0.84	$6.8*10^{-3}$	0.88	$3.8*10^{-3}$	22.10	0.37
2-feature model w/o IBN	PEP/LVET, HR		0.78	0.013	0.83	$4.6*10^{-3}$	34.32	0.58
2-feature model w/ IBN	PEP/LVET, SDRR	$\frac{x_j - \mu_{b,p}}{\mu_{b,p}}$	0.78	0.013	0.87	$3.8*10^{-3}$	23.92	0.46
2-feature model w/o IBN	PEP/LVET, SDRR		0.83	0.017	0.82	$5.8*10^{-3}$	34.53	0.73
3-feature model w/ IBN	PEP/LVET, HR, LVET	$\frac{x_j - \mu_{b,p}}{\mu_{b,p}}$	0.86	$9.5*10^{-3}$	0.90	$5.4*10^{-3}$	21.25	0.45
3-feature model w/o IBN	PEP/LVET, HR, LVET		0.92	0.018	0.84	$8.9*10^{-3}$	29.39	0.66
3-feature model w/ IBN	PEP/LVET, SDRR, LVET	$\frac{x_j - \mu_{b,p}}{\mu_{b,p}}$	0.78	0.018	0.87	$5.0*10^{-3}$	23.82	0.72
3-feature model w/o IBN	PEP/LVET, SDRR, LVET		0.78	0.011	0.85	$4.5*10^{-3}$	35.39	0.44
4-feature model w/ IBN **	HR, PEP, LVET, and PEP/LVET	$\frac{x_j - \mu_{b,p}}{\mu_{b,p}}$	0.90	$4.7*10^{-3}$	0.92	$6.0*10^{-3}$	19.47	0.12

4-feature model w/o IBN	HR, PEP, LVET, and PEP/LVET		0.81	0.047	0.85	$9.2*10^{-3}$	28.96	0.69
4-feature model w/ IBN	HR, SDRR, LVET, and PEP/LVET	$\frac{x_j - \mu_{b,p}}{\mu_{b,p}}$	0.80	$6.2 *10^{-3}$	0.89	$3.9*10^{-3}$	23.07	0.25
4-feature model w/o IBN	HR, SDRR, LVET, and PEP/LVET		0.92	$9.2 *10^{-3}$	0.87	$6.1*10^{-3}$	33.36	0.28
5-feature model w/ IBN	HR, SDRR, PEP, LVET, and PEP/LVET	$\frac{x_j - \mu_{b,p}}{\mu_{b,p}}$	0.86	$5.6 *10^{-3}$	0.91	$3.6 *10^{-3}$	21.25	0.43
5-feature model w/o IBN	HR, SDRR, PEP, LVET, and PEP/LVET		0.90	0.023	0.88	0.010	30.09	0.74
6-feature model w/ IBN	HR, SDRR, SD1/SD2, PEP, LVET, and PEP/LVET	$\frac{x_j - \mu_{b,p}}{\mu_{b,p}}$	0.84	0.016	0.89	$7.1*10^{-3}$	22.32	0.73
6-feature model w/o IBN	HR, SDRR, SD1/SD2, PEP, LVET, and PEP/LVET		0.94	0.010	0.89	$6.0*10^{-3}$	28.77	1.02
7-feature model w/ IBN	HR, SDRR, SD1/SD2, LF/HF, PEP, LVET, and PEP/LVET	$\frac{x_j - \mu_{b,p}}{\mu_{b,p}}$	0.83	$8.9*10^{-3}$	0.89	$3.5*10^{-3}$	22.73	0.47
7-feature model w/o IBN	HR, SDRR, SD1/SD2, LF/HF, PEP, LVET, and PEP/LVET		0.91	0.014	0.89	$5.0*10^{-3}$	30.22	0.63

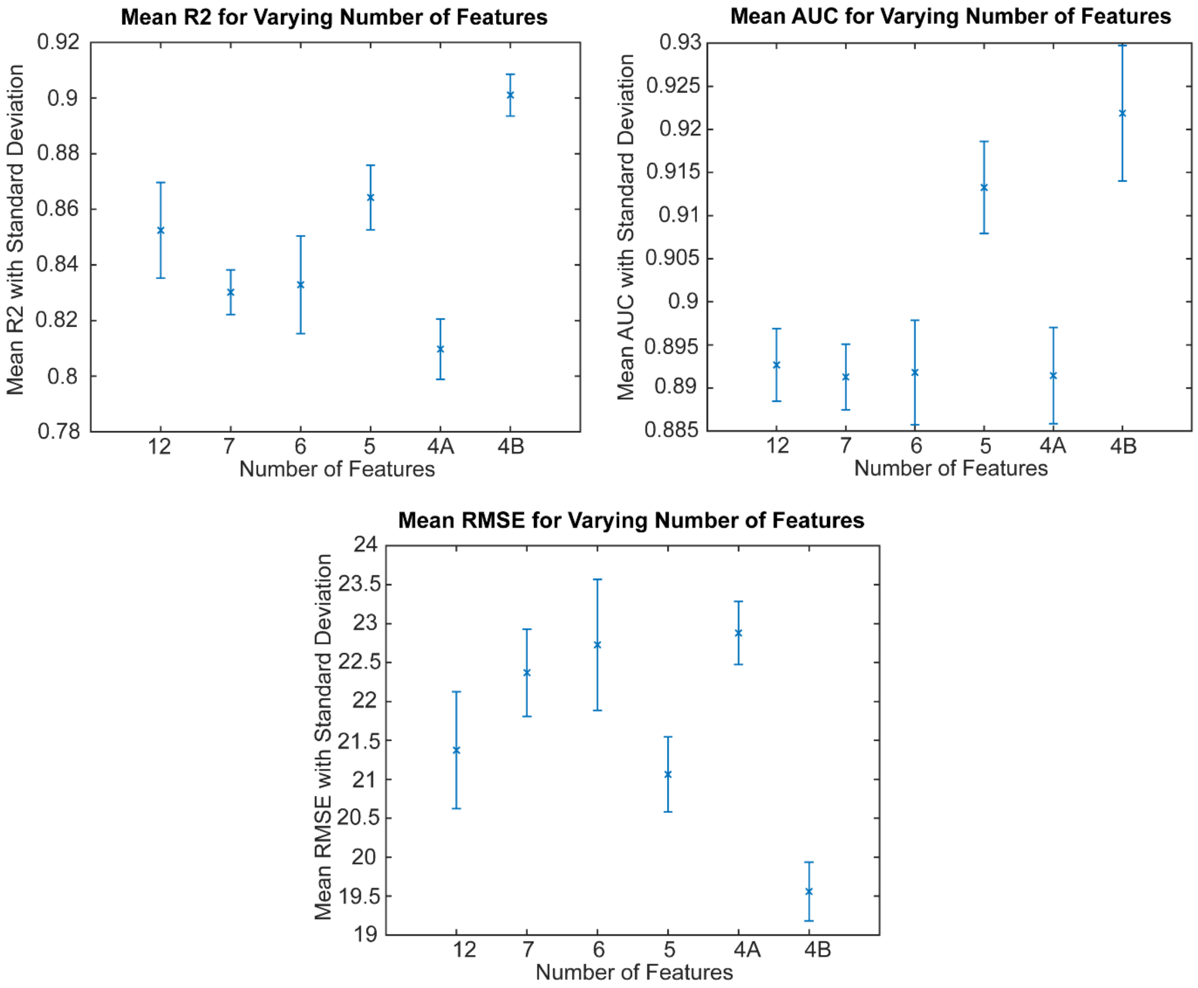

Figure S1. Graphical illustration of preliminary performance of ABVS models, including mean (M) and standard deviation (SD). "4A" represents a four-feature model containing the following features: HR, SDRR, LVET, and PEP/LVET. "4B" represents a four-feature model containing the following features: HR, PEP, LVET, and PEP/LVET.
